# The Role of Glycoprotein IIb/IIIa Inhibitors- A Promise Not Kept?

**DOI:** 10.2174/157340308784245793

**Published:** 2008-05

**Authors:** Edo Kaluski

**Affiliations:** Department of Cardiology, University Medical Center, University of Medicine and Dentistry, Newark, NJ, USA

## Abstract

For over one decade Glycoproteins IIb/IIIa inhibitors (GPI) have been administered to prevent coronary artery thrombosis. Initially these agents were used for acute coronary syndromes and subsequently as adjunctive pharmacotherapy for percutaneous coronary interventions (PCIs).

Most benefit of GPI emerged from reduction of ischemic events: mostly non-q-wave myocardial infarctions (NQWMIs) during PCI. However, individual randomized clinical trials could not demonstrate that any of these agents could significantly reduce mortality in any clinical subset of patients. Studies of employing prolonged oral GPI administration resulted in excessive death. The non-homogenous statistically-significant reduction of ischemic endpoints was accompanied by an excess of bleeding, vascular complications, and thrombocytopenia. The clinical and ecomomic burden of major bleeding and thrombocytopenia is substantial. The ACUITY trial has initiate a new debate regarding the efficacy and safety of GPI.

Selective “patient-tailored” use of GPI limited to moderate-high risk PCI patients with low bleeding propensity is suggested. Research of new algorithms emphasizing abbreviated GPI administration, careful access site and bleeding surveillance, in conjunction with lower doses of unfractionated heparin or new and safer anti-thrombins may further enhance patient safety.

## INTRODUCTION

GPI are used as adjunctive therapy for 60-70% of PCIs performed in the USA. This review attempts to summarize the current data on the efficacy and safety of these agents in PCI. In view of the data, potential modification of treatment algorithms is discussed.

## EFFICACY OF GPI (TABLE 1)

### Mortality Data

None of the individual GPI randomized clinical trials (RCTs) was ever able to demonstrate statistically-significant mortality reduction in any clinical setting. However some RCTs detected a favorable trend towards 20-30% relative risk reduction mortality in certain patient subsets. In order to prove statistically significant mortality benefit certain publications employed sub-analysis, while others have engaged in meta-analysis. The latter bring to the scientific arena complex validity concerns when performed quantitatively (due to increased risk of type I error). The need to adjust thresholds for statistical significance to account for multiple investigations or for potential heterogeneity is subject for methodological debates. Moreover, “publication bias” can further erode the meta-analysis validity: studies with unfavorable outcome are discontinued, not reported or remain unpublished, and if published are omitted from meta-analysis. As an example: 4 phase 3 RCTs of oral-GPI in ACSWSTE [1,2], which together enrolled 33,326 patients, demonstrated 31% excess mortality in the GPI arm (OR = 1.31; 95% CI: 1.12 to 1.53; P= 0.0001). Moreover the incidence of myocardial infarction increased (OR = 1.16; 95% CI: 1.03 to 1.29). These studies as well as other studies of oral GPI were not incorporated into the following meta-analysis.

The applicability and relevance GPI-RCTs on the contemporary cardiovascular arena is further diminished by certain issues (a) ***Enrollment bias:*** Certain patients were deliberately excluded from most these studies: especially patients who were considered high cardiovascular or bleeding risk (elderly, patients with heart renal or hepatic failure, history of bleeding, renal insufficiency or cerebrovascular disease). (b) ***Design bias:*** Most of the trials have carefully tracked ischemic events. The most frequently reported endpoint was post-PCI enzyme rise (CPK>2-3 times the upper limit of normal) that were named “myocardial infarctions”. The medical and economic impact of these surrogate laboratory end-points on patient outcome (like cardiovascular mortality, medical costs, length of hospital stay and patient well-being) was not usually ascertained. (c) ***Accounting for adverse events:*** Attempt to prospectively account for the clinical consequences of various drug-related adverse events (including: minor and major bleeding, vascular complications, transfusions thrombocytopenia and allergic reactions) on patients’ outcome was not diligently delineated. (d) ***Current applicability and relevance:*** From the time the studies were executed the practice of interventional cardiology has undergone considerable change: with the inception of routine versatile stenting, pre-PCI high dose (600 mg) clopidogrel, new safe and effective anti-thrombins, and optimal stenting enhanced by and pre and post-PCI lesion assessment by intravascular ultrasound and pressure wire.

As mentioned before individual GPI RCTs failed to demonstrate significant mortality reduction. The trend of reduced mortality and ischemic events was not homogenous across patient subsets and studies. In the absence of significant treatment effect on mortality in individual RCTs meta-analysis were introduced:

At 2003 a meta-analysis [3] of 12 trials (and over 20,000 **PCI** patients) demonstrated a statistically significant mortality reduction at 30 days (OR 0.73, 95% CI 0.55 to 0.96, p = 0.024 and number needed to treat to save 1 life was 357), however at 6 months this beneficial effect was diminished. (OR was 0.84 (95% confidence interval 0.69 to 1.03, p = 0.087). This meta-analysis was published 2 years after a former (2001) negative meta-analysis [4].

Meta-analysis [5] of 19 **PCI** RCTs (6 STEMI trials, the rest were ACSWSTE and elective or mixed-cohort PCI) that enrolled 20,137 patients (11,444 in abciximab studies), set the primary outcome as death at 1 and 6 months. There was a 0.47% and 0.61% absolute mortality reduction at 1 and 6 months (“number needed to treat” to save one life was 320 and 220 respectively). Mortality benefit at 6 months had a tight confidence interval (RR 0.79, 95% CI 0.64-0.97). While abciximab [6] and eptifibatide showed favorable mortality trends tirofiban had negative mortality trend. Stent placement was the initial PCI approach in only one fourth of this patient cohort. Although the authors claim the benefit was not device-specific, certain studies suggest that GPI benefit is augmented by devices like directional atherectomy [7] or balloon angioplasty [8] especially in the setting acute coronary syndromes, and less likely to affect the results of stenting or rotational atherectomy. Any myocardial infarction at 30 days was reduced by 2.3% (4.6% versus 6.9%, RR 0.63, CI 0.56-0.70). Major bleeding was significantly increased by 1.4% (4.6 versus 3.2, RR 1.26 CI 1.09-1.46). There was no accounting for minor bleeding, thrombocytopenia, vascular complications, transfusions or allergic reactions. Even when all 6 STEMI studies were pooled together no significant treatment effect of mortality was detected [9].

Meta-analysis of 6 RCTs of GPI in **ACSWSTE** [10] included 31,402 patients (18,297 on GPI) of which 24% underwent PCI at 30 days suggested that: (a) 30-day mortality (3.4% versus 3.75, p=0.14, OR 0.91 (95% CI of 0.81-1.03), and non-fatal myocardial infarction (defined as CPK rise exceeding 2-3 times the upper limit of normal) (7.4% versus 8.1%, OR=0.92, 95% CI 0.81-1.03) were not significantly reduced. (b) While Patients with elevated Troponin, ischemic ST depression on the EKG and males derived more benefit from GPI, in women (n=11,003) mortality actually increased. (c) GPI use was associated with a significant 1.1 % increase in major bleeding (1.4 versus 2.5%, OR 1.64, 95% CI 1.36-1.97).

A recent [11] multivariate and propensity analyses compared the frequency of death, reinfarction, and major bleeding during hospitalization in 38,691 patients with NSTEMI who were enrolled in the National Registry of Myocardial Infarction (NRMI)-4 (2000– 2003). Of these, 65% received GPIs only, 16.1% clopidogrel only, and 18.8% received both. The composite end point of death, reinfarction, and major bleeding was higher among patients who received both drugs rather than clopidogrel alone (odds ratio 1.31, 95% confidence interval 0.99 to 1.72).

### Ischemic Events

Most GPI studies have shown statistically significant reduction in ischemic events in the GPI arm. Most of these ischemic events were non-Q-wave myocardial infarctions (NQMIs). This beneficial effect was especially robust in high-risk patients (usually patients with ischemic ST depression, positive cardiac bio-markers or diabetics). Although, some non-RCTS [12] have claimed that even prevention of mild troponin leaks is associated with better outcome; this was never confirmed and actually contradicted by large scale RCTs: these small CPK leaks (or myocardial infarctions) have never translated into meaningful excess in 30 day or 6 months mortality. It is possible, that these “laboratory events” carry a very limited clinical impact just like the rise of troponin observed routinely after radiofrequency arrhythmia ablation or in >40% of PCIs [13].

### Other Drug Claims

Other favorable effects of GPI, like improving pre-PCI TIMI flow, or TIMI frame count [14] or improving micro-circulation perfusion in STEMI, have not translated into meaningful benefits in patient outcome. Neither abciximab nor any other GPI have ever demonstrated favorable effect on in-stent restenosis [15]or late re-intervention.

## SAFETY OF GPI

### Effects on Bleeding and Vascular Complications

Brown [16] reported FDA data on 450 deaths associated with GPI use: 44% were considered to be definitely or probably related to the use of GPI inhibitors. The author suggested that patients treated in normal clinical practice might pose a greater bleeding-risk than those treated enrolled in RCTs.

Bleeding became the most common serious complication, and among the most costly complications of PCI (incremental cost of hospitalization > $10,000, due to prolonged hospital stay and the use of additional resources). Bleeding is associated with excessive morbidity and mortality, contributing to additional treatment costs beyond those directly attributable to correcting the bleeding complication [17]. That report claims that the favorable current trend of reduced heparin dose (associated with reduced bleeding complications) is counteracted by routine administration of GPI (which results in excess of bleeding complications and thrombocytopenia).

The ACUITY [18] investigators and others, employed the concept of “net clinical benefit” which is the defined as the total number of ischemic endpoints and major bleeding events in each treatment arm. This concept is easily applicable but grossly oversimplifies and consequently distorts the true impact of the various events. One can not compare hierarchic impact of QWMI or major bleed to post-procedural CPK leak, or NQWMI. Most PCI trials reported the relative occurrence of NQWMI to QWMI in the range of 4:1 [18] to 15:1 [19]. Hence, it is possible that the clinical (not numeric) impact of bleeding events and thrombocytopenia overrides the impact of ischemic events.

The OASIS investigators [20] compared the safety and efficacy of fondaparinux versus enoxaparine in a cohort of 20,000 patients with ACSWSTE. Major and minor bleeding was associated with a relative risk of 30-day mortality of 6.5 (95% CI 5.1-8.2) and 3.0 (95% CI 2.1-4.3) respectively. Bleeding was also associated with increased incidence of stroke.

Society of Coronary Angioplasty and Interventions registry report suggests [21] that the use of GPI nearly doubles the bleeding complications. In a report by Moscucci based on data from the Global Registry of Acute Coronary Events (GRACE) registry, the overall incidence of major bleeding was 3.9% [4.8% in patients with ST elevation myocardial infarction (STEMI), 4.7% in patients with non-ST elevation myocardial infarction (NSTEMI) and 2.3% in patients with unstable angina]. Even, after adjusting for co-morbidities major bleeding was an independent predictor of in hospital death (adjusted odds ratio 1.64, 95% confidence interval 1.18, 2.28). Major bleeding occurred most frequently from gastrointestinal tract (31.5%) and vascular access site (23.8%) and was associated with an increased incidence of case fatality rate (18.6 *vs*. 5.2% p<0.001). Certain bleeding complications like retroperitoneal bleed [22], or hemorrhagic stroke [23] have a clear relation to GPI use. Predictors of bleeding are: age [24], female sex, renal dysfunction [25,26], history of bleeding, heart failure, and the use of GPI, and other invasive procedures. In some of these hemorrhagic complications, excessive dosing [27] of anti-coagulation and GPIs may have been a contributing factor.

Excess of vascular complications (which can reach 2.9%) with the use of GPI [28,29] has been reported. Predictors of vascular complications, were similar to those of bleeding complication (age, female sex, body surface area <1.6, congestive heart failure, chronic obstructive pulmonary disease, renal failure, lower extremity vascular disease, bleeding disorders, emergent priority, myocardial infarction, shock, high risk coronary lesions). Unfortunately arteriotomy closure devices have not reduced the rate or severity of vascular complications [30].

### Thrombocytopenia

Thrombocytopenia occurs fivefold more frequently with abciximab [31-33] than with the 2 other agents. Mild (<100,000), Severe (<50, 000), and profound (<20,000), occur at 4.2%, 0.7% - 1.5% and 0.4% respectively. The incidence of thrombocytopemia with eptifibatide and tirofiban is substantially lower (<0.2%). With second abciximab administration [34] thrombocytopenia is even more common (3.5-6.3%) and profound (2.0% <20,000 platelets). The duration of abciximab therapy correlates with incidence of thrombocytopenia. One center (n=300) reported the 6% thrombocytopenia with the use of abciximab [35]. Cases of abciximab induced profound thrombocytopenia occurring ≥4 days post PCI [36] are of considerable concern.

Three abciximab [37] trials demonstrated that 2.4% of 7,290 patients developed thrombocytopenia, and experienced >12-fold higher 30-day mortality rate (8.4% *vs*. 0.6%, respectively; P <0.001). Another pooled- analysis of 3 large RCTs of abciximab thrombocytopenia occurred in 3.7% (95% CI: 3.2%, 4.2%) of abciximab-treated, patients and in 1.8% (95% CI: 1.3%, 2.3%) of placebo-treated patients (p < 0.001). Patients with thrombocytopenia had significantly higher rates of major bleeding, major hemoglobin reduction and increased transfusion requirements of both blood and platelets compared with those without thrombocytopenia.

In a retrospective analysis [38] of the CADILLAC data, 50 of 1,975 qualifying patients (2.5%) who underwent primary PCI acquired thrombocytopenia. Patients who developed thrombocytopenia had higher in-hospital rates of major hemorrhagic complications (10.0% versus 2.7%, p = 0.01), greater requirement for blood transfusions (10.0% versus 3.9%, p = 0.05), longer hospital stay (median 4.8 versus 3.6 days, p = 0.008), and increased costs (median dollar 14,466 versus dollar 11,629, p = 0.001). All-cause mortality was markedly increased at 30 days (8.0% versus 1.6%, p = 0.0008) and at 1 year (10.0% versus 3.9%, p = 0.03). Thrombocytopenia [39] tends to at-least double the hospital stay.

Patients who developed thrombocytopenia during treatment with orbofiban (0.92% of the treated cohort) had higher rates of death (11.6% *vs*. 1.7%, p < 0.001), recurrent MI (12.1% *vs*. 2.8%, p < 0.001), intracranial hemorrhage (2.9% *vs*. 0.0%, p < 0.001), and major or severe bleeding (19.0% *vs*. 2.0%, p < 0.001) at 30 days [40].

## RECENT TRIALS

### *ACUITY* [18] Trial

ACUITY (Acute Catheterization and Early Intervention Triage Strategy) assessed safety and efficacy of PCI in 13,819 patients with ACSWSTE using three pharmacotherapy strategies: bivalirudin alone, bivalirudin with GPI, and heparin or enoxaparine with GPI. In 9,207 who were assigned to GPI mortality benefit was not observed. Moreover, there was no reduction in ischemic events, NQWMI or QWMI in the GPI arms. The arm receiving bivalirudin reported 3% major and 12.8% minor bleeding. Incidence of major and minor bleeding escalated to 5.3% and 21.7% respectively when GPI was added to bivalirudin. Similar bleeding rates (5.7% and 21.6% respectively) were reported when GPI was added to heparin or enoxaparine. Importantly, the duration of GPI administration was relatively abbreviated prior to PCI (5 hours). The lack of benefit of GPI before PCI or after PCI was even more pronounced in subset patients with pre-PCI treatment of clopidogrel.

### *STEEPLE *[41] Trial

This trial enrolled 3528 patients undergoing non-emergent femoral access PCI. The study excluded patients who were at higher risk for bleeding complications. Patients were assigned to 2 enoxaparine arms (0.5mg/k and 0.75 mg/kg) or heparin arm. In all study arms, major and minor bleeding events nearly doubled with the use of GPI (from 4.1%, 3.6% and 6.8% to 8.6%, 10.8% and 11.2% respectively.

## COST EFFECTIVENESS

Since homogenous efficacy is conditional and germane to any cost-effective analysis, cost effectiveness is extremely difficult to ascertain. Most cost-effectiveness studies have not determined the cost of mortality reduction. Instead these studies focused on preventing merely ischemic events [42,43] or combination of ischemic events and mortality[44] and have not disclosed the entire spectrum of adverse events of GPI on clinical outcome[45]. The health benefits of reducing CPK leaks or NQWMI is difficult to define. These reports may have miscalculated the impact of NQWMIs, major and minor bleeding events, and thrombocytopenia when assessing the “net clinical benefit”. A favorable “net-clinical benefit” can probably be demonstrated for high-risk PCI patients, without clopidogrel pre-PCI loading, who have low bleeding and thrombocytopenia propensity. Recent cost-analysis reports suggest that the mere use of bivalirudin may render GPI a surplus [46-48].

## CURRENT PCI GUIDELINES

### Elective PCI

The European guidelines [49] suggest that GPI should not be routinely used for PCI of stable angina pectoris and should be applied selectively to patients who undergo complex or high risk PCI. The American guidelines [50] state that it is reasonable to use GPI in stable coronary syndromes [Class IIa recommendation, level of evidence (LOE) B].

### Acute Coronary Syndromes Without ST Elevation (ACSWSTE)

The European guidelines suggest that GPI (tirofiban and eptifibatide) could be given prior to angiography (upstream) to high risk patients with ACSWSTE who are subject to early “invasive approach” (early cardiac catheterization and revascularization). These guidelines also suggest that GPI (abciximab and eptifibatide) can be given in the cardiac catheterization laboratory if angiography is planned within <2.5 hours. The American guidelines suggest (class I recommendation LOE-A) that all patients with ACSWSTE undergoing PCI without clopidogrel, should receive GPI (abciximab, tirofiban or eptifibatide); while those who have received clopidogrel should be considered for GPI (Class IIa, LOE-B).

### In ST Elevation MI (STEMI)

Abciximab (Class IIa, LOE-B) or tirofiban or eptifibatide (Class IIb LOE-C) are optional by both the American and European guidelines.

The European guidelines also endorse Bivalirudin as the anti-thrombin of choice whether or not GPI are given.

The use of GPI for the following indications is discouraged:

1. Adjunctive therapy for fibrinolysis in acute myocardial infarction
2. Adjunctive therapy to patients with ACS without ST-elevation who are treated with medical therapy, and are not candidates for PCI.
3. Primary or secondary prevention [51].

Although there are considerable similarities between the European and the American guidelines there is profound difference between the use of GPI during PCI in the USA (68% according to the ACC registry) and Europe (23% according to the 2006 Euro-Heart Survey).

## CHANGES IN TREATMENT STRATEGY

### Should we Use it at all (Table 2)?

In view of current current data, the answer to this question is not at all clear. Since the relative ischemic benefits should clearly outweigh the bleeding risk to justify the decision to initiate or continue GPI therapy. The patients who benefit the most from GPI are patients identified as “high risk” by currently available risk scales ACSWSTE [52], STEMI, cardiogenic shock [53], or complex elective PCI [54]. Also at risk are diabetics [55-57], patients with chronic renal insufficiency and heart failure. GPI do not seem to favorably affect PCI of degenerated vein grafts [58,59], or rotational atherectomy [60]. Female patients derive very little benefit from GPI [61].

Patients who are at high risk of bleeding (elderly, chronic renal failure, known bleeding pathology, heart failure, females [61], or anemia of unknown cause) should avoid GPI or minimize drug exposure time.

Clopidogrel pre-PCI treatment [62], by reducing periprocedural NQWMI, can potentially minimize the incremental benefit of GPI [63]. Indeed for elective low-to-intermediate risk PCI, after pretreatment with 600 mg of clopidogrel, abciximab was associated with no measurable clinically benefit within the first 30 days after PCI [64]. However patients with acute coronary syndromes of moderate to high risk still derived some benefit from abciximab when superimposed on loading dose (600 mg) of clopidogrel [65].

### Routine Upstream Use?

In the ACUITY trial [66] routine administration of upstream GPI in a moderate to high risk cohort of ACSWSTE undergoing PCI when compared to in-lab administration resulted in excessive GPI use (98.3% versus 55.7%) longer duration of administration (median, 18.3 versus 13.1 hours; P<.001) excessive 30-day rates of major bleeding (6.1% versus 4.9% P<.001) and mild reduction of ischemic end points (7.1% *vs*. 7.9%, relative risk 1.12; 95% confidence interval, 0.97-1.29; P = .044) and similar rates of net clinical outcomes (11.7% *vs*. 11.7%; P<.001 for noninferiority; P = .93 for superiority). The authors concluded that routine upstream GPI use is not justified in this cohort of patients.

In STEMI a meta-analysis of 6 randomized trials, early (upstream) administration of GPI in STEMI appeared to improve coronary patency with favorable trends for clinical outcomes [67]. However the favorable effect of upstream administration on mortality or meaningful clinical events, did not reach statistical or clinical significance.

Even with upstream GPI and invasive approach the TIMI -18 –TACTICS investigators [68] were not able to demonstrate mortality benefit at 30 days (1.6 *vs*. 2.2, p=0.29) or at 6 months (3.5 *vs*. 3.3%, p= 0.74).

### Which drug ?

Although comparative analysis between trial results [4], and certain trials suggested superiority of one agent over the other [69] this advantage did not translate into a meaningful superiority of any agent in a head-to-head randomized controlled trial [70-72]. The advantages of Integrilin over abciximab are:

1. Better safety profile: lower rate of allergic reactions, thrombocytopenia [35]
2. Reduced cost (especially if used as bolus only results in considerable savings)
3. Effects of the drug are terminated faster (shorter biologic half life)
4. The drug can be re-administered without safety or efficacy issues.
5. Ease of use (no specific filter)

The advantage of Integrilin over Tirofiban is more extensive experience for in-lab use in RCTs. Some studies suggest that with current dosing more patients achieve “target” platelet inhibition [73,74] with integrilin or abciximab than with tirofiban.

### The Dose

Bleeding events tend to correlate more with the duration of administration of GPI and anti-coagulation and with the intensity of anti-coagulation [75] than with the dose of GPI. One retrospective study suggested that GPI overdosing may be a contributor to bleeding complications [27]. Short term use of high doses of GPI [76] or inadvertent iatrogenic overdose is rarely associated with toxicity or bleeding. Intracoronary administration of eptifibatide [77] and abciximab have been described as safe, however the advantage of this method over conventional intra-venous administration is not proven. Studies using both intra-venous [78] and oral [1] prolonged administration demonstrate unfavorable clinical outcome.

The GOLD study reported correlation between insufficient platelet inhibition and excess of ischemic events. However, the unresponsive arm of GOLD had event rates that far exceeded event rates without GPI in most RCTs [79]. The preferred way to assess platelet inhibition, target value of platelet inhibition, and algorithms for dose adjustment have never been widely assessed, confirmed, and applied to clinical practice. Moreover, the correlation between the extent of platelet inhibition and clinical efficacy are very loosely associated [80].

### Dose and Duration of Anticoagulation

PCI has advanced to the stage the major ischemic complications are rare and related to suboptimal patient and lesion selection, procedural technique and procedural results. The effects of the dosage and brand of anti-coagulant on ischemic complications are not scientifically compelling. However the decision whether to use an anticoagulant, at what dose and for what duration have far-reaching consequences on bleeding complications.

There is emerging body of evidence suggesting that PCI can be executed with low dose heparin [81] or no heparin at all [82]. A recent RCT study (CIAO presented in TCT 2006 by Stabile *et al.*) showed that PCI can be performed safely with no heparin or GPI. In patients with high bleeding propensity low doses of short acting anti-thrombin agents should be used [83].

### The Decision to Administer GPI Post PCI

The duration of GPI administration and anti-coagulation, correlates with both bleeding complications and costs. The optimal duration for post PCI administration was never subject to rigorous trials. However certain randomized single center trials were able to demonstrate similar efficacy and better safety when eptifibatide [84] tirofiban [85,86] or abciximab [87] were given as a single bolus or discontinued post PCI. Many physicians believe that post- PCI GPI administration should we carefully assessed in view of the procedural result, the state of access sites, and patients’ bleeding propensity. Since access site bleeding along with gastrointestinal bleeding are the most frequently encountered bleeding sites, these sites should be carefully inspected for bleeding. The physician must understand that timely discontinuation of GPI is crucial in a bleeding patient. By enlarge being cautious and employing conservative in post-PCI GPI administration is advised for patients with intermediate-high bleeding propensity.

## SUMMARY

In view of questionable mortality benefit, and evidence suggesting reduced ischemic events but excessive bleeding vascular and thrombocytopenia complications the clinician should carefully and individually decide on the use GPI along with other adjunctive pharmacotherapy. Decision regarding when to initiate and terminate the GPI therapy, at what dose, should be based on individual patient risk versus benefit.

## Figures and Tables

**Table 1:** GPI RCTs- Effect on 1-6 Months Mortality

Meta-Analysis or RCT	Patient Population	P	Absolute Risk Reduction (%)	Relative Risk (95% CI)
Karvouni [5] (19 PCI RCTsθ) 6 months	20,137 Mixed PCI (STEMI*, ACSWSTEφ Elective)	0.028-0.048	0.55	O.79 (0.64-0.97)
Boersma [10] (6 ACSWSTE RCTsθ) 30 days	31,402 ACSWSTEφ (only 24% PCI)	0.14	0.3	0.91 (0.81-1.03)
ACUITY [18] (ACSWSTEφ) 30 days	13,819 ACSWSTEφ 99% Angiography	NS	0.2	0.875
Oral GPI [1,2] (4 RCTsθ ACSWSTEφ and PCI) 30 days	26,094 ACSWSTEφ 7232 PCI	0.001	-0.4	1.31 (1.12-1.53)

φ ACSWSTE- acute coronary syndrome without ST elevation.

* STEMI- ST elevation myocardial infarction

θ RCTs – Randomized clinical trials

**Table 2. GPI Administration Based on Non-Validated Bleeding Propensity Score:** (Bleeding score:low<3;        moderate 3-5;high risk≥6) Age>80 – 3 points          Recent (<3 months) bleeding- 6 points Age>65- 2 point           Remote (>3months) bleeding or previous PCI bleed– 2 points Creatinine>2mg/dl- 2 points      Compensated heart failure – 1 points Dialysis- 3 points          Anemia Hemoglobin <10- 3 points Female- 1 point           Anemia Hemoglobin<8- 6 points

Bleeding Propensity	Low risk Elective PCI	High risk or Complex Elective PCI	ACSWSTE φ	STEMI *
Low Bleeding score (<3 points)	35 -70 u/kg UFH or Bivilirudin	35-70 u/kg UFH GPI or Bivilirudin	70 u/kg UFH GPI or Bivilirudin	70 u/kg UFH GPI or Bivilirudin
Moderate bleeding score (3-5 points)	35 u/kg UFH \ or Bivilirudin	35 u/kg UFH GPI^ or Bivilirudin	35 u/kg UFH GPI^ or Bivilirudin	70 u/kg UFH GPI^ or Bivilirudin
High bleeding score (>5 points)	35 u/kg UFH or Bivilirudin	35 u/kg UFH or Bivilirudin	35 u/kg UFH or Bivilirudin	70 u/kg UFH or Bivilirudin

φ ACSWSTE- acute coronary syndrome without ST elevation.

* STEMI- ST elevation myocardial infarction (May change with the results of HORIZON-AMI study)

Ф Non-validated bleeding propensity score.

^ Abbreviated (bolus only) GP IIb/IIIa-B administration should be considered if PCI yields satisfactory angiographic and clinical results.
